# Uric Acid as a Marker of Mortality and Morbidity in Fabry Disease

**DOI:** 10.1371/journal.pone.0166290

**Published:** 2016-11-11

**Authors:** Daniel Rob, Josef Marek, Gabriela Dostálová, Lubor Goláň, Aleš Linhart

**Affiliations:** 2nd Department of Medicine - Department of Cardiovascular Medicine, First Faculty of Medicine, Charles University and General University Hospital, Prague, Czech Republic; Cincinnati Children's Hospital Medical Center, UNITED STATES

## Abstract

**Background:**

Serum uric acid (UA) elevation is common in patients with cardiovascular, renal and metabolic diseases. However, no study to date has analysed the role of UA in Fabry disease (FD).

**Objectives:**

To evaluate the association between serum UA levels and mortality and morbidity in FD.

**Materials and Methods:**

We conducted a post-hoc analysis of a prospectively followed-up cohort of 124 patients with genetically proven FD. Serum UA levels were acquired at baseline; clinical events and mortality were assessed during regular visits every 6 to 12 months. The primary endpoint was a composite of multiple secondary outcomes: all-cause mortality, adverse cardiovascular events, progression of renal dysfunction and stroke or transient ischaemic attack (TIA). Predictive value was assessed using the Cox proportional hazards model and the Kaplan Meyer estimator.

**Results:**

During follow-up of 7.4 ± 3.7 years, 64 (52%) patients reached the primary combined endpoint. Overall, UA levels were significantly associated with combined outcome (p < 0.001) and remained independently associated after correcting for age, sex and estimated glomerular filtration rate (hazard ratio [HR] per 20 μmol/l increase 1.09, 95% confidence interval [95%CI] (1.00–1.19), p = 0.04). UA was associated with overall mortality in univariate analysis (p = 0.021); however, the association did not reach statistical significance after multivariate correction (HR per 20 μmol/l increase 1.07 95%CI 0.93–1.25, p = 0.32). Higher UA levels were also associated with cardiac adverse outcomes, progression of left ventricular hypertrophy and progression of renal dysfunction (ps < 0.001). No association was observed between UA levels and stroke or TIA (p = 0.323).

**Conclusion and Implications:**

Increased serum UA levels may represent an independent risk factor for adverse clinical outcomes in Fabry patients and are associated with all-cause mortality. UA is a widely available and cheap biomarker that may improve risk stratification of Fabry patients in clinical practice.

## Introduction

Fabry disease (FD) (OMIM 301500) is an X-linked lysosomal storage disorder caused by α-galactosidase A (agalA) gene mutations that result in a decrease or absence of agalA enzymatic activity. This results in progressive accumulation of neutral glycosphingolipids in various tissues [1]. The clinical manifestations of FD depend on sex, age, and mutation type. Males with the classic form of FD suffer from cutaneous lesions (angiokeratomas), hypohidrosis, neuropathic pain, cardiomyopathy, renal function impairment, and premature cerebrovascular complications. Extensive variation in the severity of symptoms and complications occurs even within the same family. Therefore, finding an easily available and inexpensive marker that could identify persons at risk of developing complications is of considerable importance in the clinical management of patients [2].

Uric acid (UA) is the metabolic end product of purine metabolism in humans. UA elevation is very common in patients with cardiovascular, renal and metabolic diseases [3]. Increasing evidence based on experimental and clinical studies suggests that UA levels reflect the degree of oxidative stress, inflammation and endothelial dysfunction and are a predictor of morbidity and mortality in cardiovascular, renal and metabolic disorders [3–5]. Therefore, we evaluated the potential predictive role of UA levels on the risk of mortality and morbidity in our cohort of patients with genetically proven FD.

## Materials and Methods

### Study design

Informed written consent was obtained from patients for their clinical follow-up and included an agreement with processing and analysis of anonymised clinical data for scientific purposes. The research was approved by the Ethics Committee of the General Faculty Hospital and First Medical Faculty, Charles University, Prague. Clinical investigation have been conducted according to the principles expressed in the Declaration of Helsinki. We conducted a post-hoc analysis of a prospective cohort of patients with genetically proven FD who were followed-up in the National Referral Centre for FD of the General University Hospital in Prague. This prospective cohort has been continuously recruited since 1996 with the systematic recording of clinical events and analysis of clinical data. The current data has been collected during the period 2000 and 2015 from patients older than 18 years and were analysed locally. Data of our patients are also contributing to large international registries (Fabry registry, Fabry Outcome Survey).

### Study population

We included all adult FD patients diagnosed in our centre from 2000 to 2015 that had baseline UA assessment and at least one follow-up visit. The diagnosis of FD was based on demonstrated reduction of α-Gal A activity in leukocytes or plasma and confirmed by DNA mutation analysis. The study population consisted of 124 patients. Five patients were excluded from the final analysis because of missing follow-up data. Of these five patients, three were lost to follow-up and two were diagnosed during the last year of the recruitment period and as yet did not have any follow-up visit. Thus, the final study population comprised 119 patients.

### Determination of clinical and laboratory data

All blood specimens were processed by the clinical laboratory at the General University Hospital in Prague. Determination of serum UA concentration was done by the uricase/peroxidase enzymatic photometric method using a modular analytics automatic analyser (Roche Diagnostics, Basel Switzerland). All other characteristics, including clinical history, electrocardiogram, echocardiography, urinalysis and biochemistry were obtained at the first visit, at each routine follow-up visit (intervals between the visits ranged from 6 to 12 months) and at any additional clinical evaluation required because of the patient’s condition. The estimated glomerular filtration rate (eGFR) was calculated using the CKD-EPI (Chronic Kidney Disease Epidemiology Collaboration) equation [6]. Left ventricular mass index (LVMi) was calculated using the Devereux-modified cube formula based on linear measurements of interventricular septum thickness, left ventricular cavity diameter and posterior wall thickness as assessed by the accelerated solvent extraction (ASE) method and indexed to body surface area (in m^2^) [7]. Data regarding initiation, duration and compliance with the ERT therapy has been tracked regularly during the visits. Although currently, our center is complying with the internationally recommended principles suggesting that ERT should be given to all patients at earliest appearance of relevant clinical signs and symptoms [8,9], these principles were not applicable in the past due to reimbursement limitations in the Czech Republic. Therefore indications for ERT were changing over time and patients with clinical complications were prioritized.

### Outcome assessment

The primary endpoint was a composite of multiple secondary outcomes: (1) all-cause mortality, (2) cardiac complications, including angina pectoris CCS II-IV, acute myocardial infarction, heart failure NYHA II-IV, syncope, clinically relevant arrhythmias, implantable cardioverter defibrillator (ICD) or pacemaker implantation and (2b) progression of left ventricular hypertrophy defined as an increase in LVMi by 30 g/m^2^, (3) progression of renal disease defined as a decrease in the eGFR rate by 10 mL/min/1.73 m^2^ or progression to end-stage renal disease requiring renal replacement therapy or (4) stroke or transitory ischaemic attack (TIA). Separate analyses for each of the subgroups were performed as secondary endpoints.

### Statistical analysis

Continuous variables are reported using mean ± standard deviation (SD) as well as quartiles (25^th^, 50^th^ and 75^th^ percentile). Categorical variables are reported as proportions. Normality of baseline variables was assessed using Shapiro-Wilks test. Because baseline continuous variables didn’t follow normal distribution, the Mann-Whitney U test was performed to compare continuous variables and the Chi-square test to compare categorical variables in baseline characteristics.

The Kaplan-Meier estimator with the log-rank test was performed to assess differences in event rates among different levels of UA, both overall and for pairwise comparisons. For purposes of the Kaplan-Meier analysis, the population was divided according to UA tertiles. Cox proportional hazards model was used for calculation of hazard ratios (HR) with confidence intervals. Multivariate analysis was done using a Cox proportional hazards model with UA as a continuous variable. In multivariate modelling we have adjusted levels of uric acid to established prognostic factors in Fabry disease, i.e. gender, age and renal function. Due to limited number of outcomes we included 4 total variables in the model to avoid overfitting. The proportional hazards assumption was tested using time-dependent covariates and was found to be adequate.

All statistical analyses were done using the R software version 3.2.3 (R Foundation for Statistical Computing, Vienna, Austria). A p-value of < 0.05 was considered significant.

## Results

### Baseline characteristics and concomitant therapy

The evaluable population consisted of 119 patients. The average length of follow-up was 7.4 ± 3.7 years. Baseline demographic characteristics according to the presence or absence of the primary endpoint are listed in Table 1. Patients who experienced clinical events were more frequently male, older, presented a higher baseline LVMi and serum creatinine, have lower eGFR and higher UA levels. At baseline and during follow-up, patients who experienced clinical events more frequently received enzyme replacement therapy (ERT) for a longer period of time. Table 2 shows the concomitant therapy of the two groups. At baseline, the concomitant therapy did not differ between the groups and only two patients received low-dose allopurinol (100 mg daily) before study enrolment. During follow-up, the adjunctive therapy increased in both groups but more so in patients with clinical events. At baseline, more men were on renal replacement therapy as compared to women (22% vs 4%) and also had higher incidence of arterial hypertension (46% vs 26%). Otherwise there were no significant differences in baseline characteristics between the genders.

**Table 1:** Baseline characteristics.

Variable	No event		Adverse event		P-value
	(N = 55)		(N = 64)		
	25%/median/75%	Mean ± SD	25%/median/75%	Mean ± SD	
Age (years)	25/36/52	38 ± 15	31/46/54	44 ± 14	0.032
Uric acid (μmol/L)	216/258/299	264 ± 68	244/301/383	319 ± 97	0.001
Estimated GFR (ml/min)	80/97/105	91± 27	51/ 77/104	75 ± 34	0.013
Creatinine (μmol/L)	65/74/84	100 ± 113	75/ 88/126	141 ± 156	<0.001
LVMi (g/BSA)	65/76/119	92 ± 35	88/131/166	143 ± 80	<0.001
ERT duration (average of all), (years)	0.00/0.00/0.00	1.05 ± 2.60	0.36/5.82/10.84	5.72 ± 4.74	<0.001
ERT duration (treated only, n = 61), (years)	1.9/2.6/7.5	4.5 ± 3.7	4.6/7.7/11.2	7.6 ± 3.9	0.012
Female sex	76% (42)		48% (31)		0.002
Arterial hypertension	25% (15)		39% (25)		0.094
Diabetes	7% (4)		9% (6)		0.58
Smoking	12% (7)		25% (16)		0.056
Transplant/dialysis	5% (3)		16% (10)		0.054

GFR, Glomerular Filtration Rate; LVMi, Left Ventricular Mass index; BSA, Body Surface Area

**Table 2:** Concomitant medication.

	At baseline			At last follow-up		
Medication	No event (n = 55)	Adverse event (n = 64)	P-value	No event (n = 55)	Adverse event (n = 64)	P-value
ERT	1 (1.8%)	8 (12.5%)	0.028	11 (20%)	43 (67%)	< 0.001
ACE inhibitor/AT blocker	0 (0.0%)	1 (1.6%)	0.352	12 (22%)	39 (61%)	< 0.001
Beta-blocker	0 (0.0%)	1 (1.6%)	0.352	10 (18%)	27 (42%)	0.005
Calcium channel Antagonist	0 (0.0%)	0 (0.0%)	-	4 (7.3%)	25 (39.1%)	<0.001
Allopurinol	0 (0.0%)	2 (3.1%)	0.186	1 (1.8%)	15 (23.4%)	<0.001
Antiplatelet	0 (0.0%)	1 (1.6%)	0.352	5 (9.1%)	31 (48.4%)	<0.001
Anticoagulation	0 (0.0%)	1 (1.6%)	0.352	1 (1.8%)	12 (18.8%)	0.003
Diuretics	0 (0.0%)	0 (0.0%)	-	6 (11%)	20 (31%)	0.007

ERT, Enzyme Replacement Therapy; ACE, Angiotensin Converting Enzyme; AT Angiotensin

### Event analysis according to serum uric acid levels

**Fig 1:**
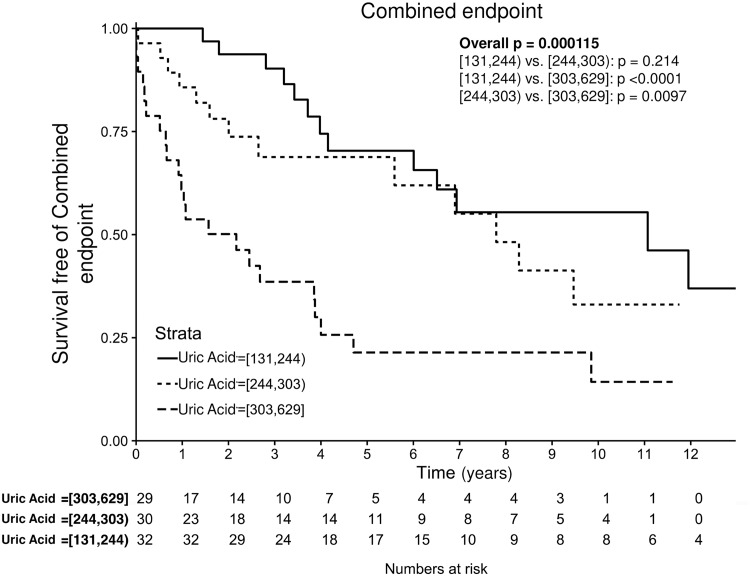
Kaplan-Meier analysis of combined endpoint according to tertiles of UA. Patients with higher UA levels had significantly worse primary outcome. This result remained independently associated after correcting for age, gender and estimated GFR (p = 0.04).

**Fig 2:**
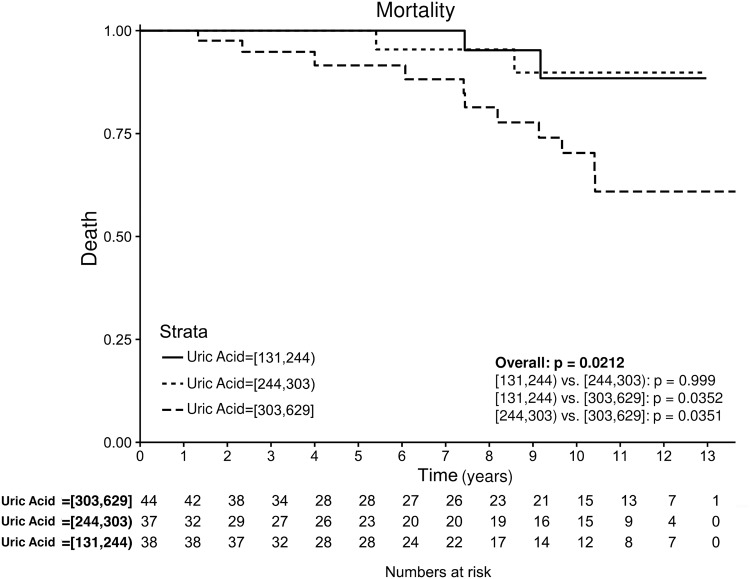
Kaplan-Meier analysis of all-cause mortality according to tertiles of UA. Patients with higher UA levels had significantly UA increased overall mortality in univariate analysis (p = 0.021).

During the follow-up, 64/119 patients had reached the primary endpoint. Patients with higher UA levels had a significantly worse primary outcome (Fig 1, p < 0.001). Furthermore, UA retained independent predictive power for primary outcome after adjusting for age, sex and renal function (hazard ratio [HR] per 20 μmol/l increase 1.09, 95% confidence interval [95%CI] 1.00–1.19, p = 0.040, Table 3). Levels of UA were also associated with overall mortality in univariate analysis (p = 0.021, Fig 2), but they did not reach statistical significance after correcting for the covariates (HR per 20 μmol/l increase 1.07 95%CI 0.93–1.25, p = 0.32).

**Table 3:** Multivariate model.

Variable	HR (95%CI)	Significance
Uric acid (per 20umol/l)	1.09 (1.00–1.19)	0.040
eGRF (per 10ml/min/1.72m^2^)	0.92 (0.78–1.08)	0.311
Age (per 10 years)	1.21 (0.92–1.60)	0.171
Female sex	0.44 (0.22–0.86)	0.017

GFR, Glomerular Filtration Rate

**Fig 3:**
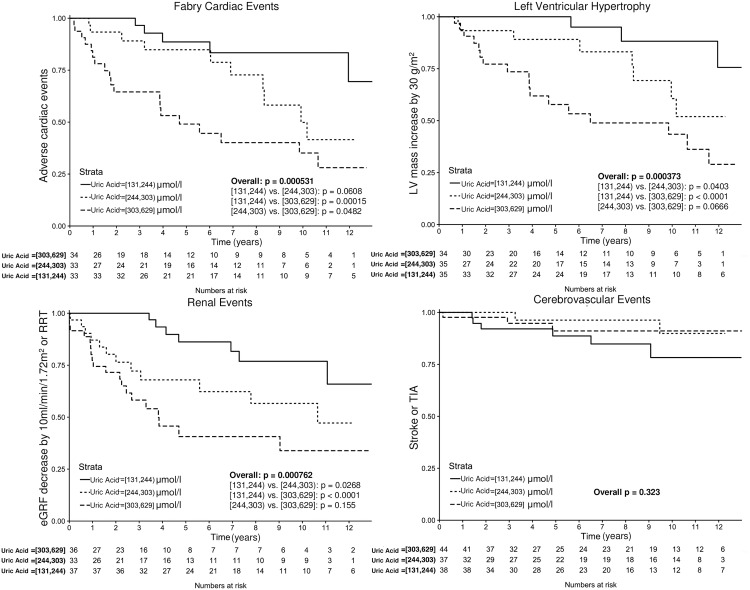
Kaplan-Meier analysis according to tertiles of UA for A) Fabry cardiac events B) progression of left ventricular hypertrophy C) renal events (p < 0.001 for all), and D) cerebrovascular events where no significant association was observed (p = 0.323).

When analysing organ-specific secondary endpoints, higher UA levels were associated with cardiac adverse outcomes (p < 0.001, Fig 3a). More specifically, the patients with the highest tertile had significantly worse outcome than those with the lowest tertile (HR 5.54, 95%CI 2.06–14.9, p < 0.001). Furthermore, progression or development of left ventricular hypertrophy, when analysed separately, was also associated with UA levels (p < 0.001, Fig 3b), with a HR of 8.01, 95%CI 2.35–27.7, p < 0.001 at the highest tertile compared with the first. Similarly, progression of renal dysfunction was associated with UA levels (p < 0.001, Fig 3c), with the highest tertile of UA having the worst outcome (HR vs. first tertile 4.75, 95%CI 1.98–11.36, p < 0.001). No significant association was observed between UA levels and cerebrovascular complications (p = 0.323, Fig 3d).

## Discussion

To our knowledge, this is the first study suggesting a potential role of UA as a predictor of clinical outcomes in FD. Of note, the predictive role of a baseline UA level for the primary composite endpoint remained significant even when age, sex and baseline renal function were taken into account. Therefore, it seems plausible to hypothesise that UA is not merely a marker reflecting global oxidative stress but may also play an independent pathophysiological role in the progression of FD.

The exact underlying mechanisms by which UA may influence FD progression have not yet been analysed. However, the mechanisms of UA production and metabolism may interfere with the disease course at different levels. On the other hand, it is plausible that several FD complications and therapies directly or indirectly increase UA levels.

### The role of xanthin oxidase (XO) and oxidative stress

UA represents the end-product of purine metabolism in humans [10]. The xanthin oxidase (XO)–xanthine dehydrogenase (XDO) enzymatic complex has a key role in UA production. This complex catalyses the oxidation of hypoxanthine to xanthine and further catalyses the oxidation of xanthine to UA [11]. For its ability to generate reactive oxygen species (ROS), XO is considered one of the most powerful oxygen radical–generating systems in human physiology, seconded by NADPH oxidase pathways [11–14]. It should be noted that two of the tissues with the highest XO activity are the capillary endothelium and the endothelium of the small arteries [15]. Thus, while a direct pathogenic role for UA in the vasculature remains unproven, its presence at elevated concentrations seems to indicate increased XO activity, oxidative stress and consequent endothelial damage [16,17]. The causal role of XO in the pathophysiology of vascular damage is substantiated by the observation that inhibition of harmfully increased XO activity leads to improvement across a range of surrogate markers in patients with cardiovascular and renal disorders [18–20]. Several studies have shown an association between increased UA concentrations and oxidative stress [21–23], endothelial dysfunction [24–26] and inflammation [27,28]. All of these adverse effects are thought to have a role in lysosomal storage diseases (LSDs), including FD [29]. Above all, ROS seems to play a key role in inducing pro-apoptotic processes in LSDs [29]. In FD, Shen et al. have shown that excess intracellular globotriaosylceramide induces oxidative stress [30]. In addition, several other studies have demonstrated the role of oxidative stress in the pathogenesis of FD [31,32]. Furthermore, progressive storage of glycosphingolipids leads to activation of signalling pathways that result in hypertrophy, apoptosis, necrosis, inflammation and fibrosis [33], pathways that are strongly related to oxidative stress. Another important phenomenon that occurs in FD is abnormal energy metabolism [34,35]. Current data show that XO activity and UA levels are increased in the settings of abnormal energy metabolism in various disorders [36,37]. In conclusion, it seems that increased UA levels in Fabry patients are caused by enhanced XO activity and ROS production that may promote pathophysiological changes and disease progression.

### The role of endothelial dysfunction

Oxidative stress holds an important role in the pathogenesis of cardiovascular disease largely through its contribution to endothelial dysfunction [38]. A number of studies have shown that elevated serum UA may play a role in the development of endothelial dysfunction [24,39,40]. Significance of XO-induced endothelial dysfunction is further supported by studies in which treatment with higher doses of allopurinol led to improved endothelial function [41–44]. Endothelial dysfunction is apparently a feature frequently seen in FD and appears to be closely linked to elevated levels of myeloperoxidase, excessive production of ROS and endothelial nitric oxide synthase uncoupling [45,46].

### The role of inflammation

A positive and significant association between UA and inflammatory markers has been reported in several studies [27,47]. The pro-inflammatory state participates in the pathogenesis of FD [48] and the inflammatory cytokines IL-6 and TNF-α are both significantly increased in Fabry patients [32].

### The role of comorbidities

From a clinical perspective, our results suggest that elevated UA levels may represent an independent biomarker of cardiac, renal and metabolic risk in Fabry patients. Cardiovascular involvement in FD contributes substantially to disease-related morbidity and mortality [49]. Chronic kidney disease is a prominent feature of FD [50]. Our findings are in concordance with a number of studies showing that UA level is a predictor of morbidity and mortality in coronary artery disease [51], heart failure [52], hypertrophic cardiomyopathy [53] and dilated cardiomyopathy [54]. UA has also been incriminated in several cardiovascular, renal and metabolic disease states that frequently occur in Fabry patients, including hypertension [55,56], left ventricular hypertrophy [57], stroke [58], kidney disease [59–61], diabetes and metabolic syndrome [62,63]. Therefore, the elevation of serum UA observed in our cohort reflects cardiovascular, renal and metabolic risk of Fabry patients. Of note, UA monitoring cannot replace cardiac and kidney surveillance in FD. However UA can be used as an additive tool for risk assessment and elevated UA levels should represent an argument for closer patient surveillance.

### The role of concomitant therapy

It should be acknowledged that several drugs may have an impact on baseline UA levels. Among them, diuretics cause an important increase in UA levels. To a lesser extent small doses of acetylsalicylic acid and beta-blockers may induce an increase in UA levels as well. However, only a minority of patients received these drugs at baseline. The same applies to allopurinol, the only XO inhibitor used in the Czech Republic during the course of the study. During the clinical follow-up, several patients received this treatment, which apparently did not influence the predictive value of baseline UA levels. The influence of ERT therapy on UA levels as well as the potential role of UA in ERT therapy guiding was not addressed in our study and should be evaluated in future studies.

### Strengths and limitations

The Czech Republic, with a population of approximately 10.5 million inhabitants, has a single centre for FD. This situation allows us to provide detailed clinical and biochemical records of a substantial group of Fabry patients. Strength of our results are also supported by long-term follow-up of Fabry patients (average 7.4 years) and systematic measurements of UA during routine visits. This study has several limitations. First, our observations are based on post-hoc single-centre observational data. Multicentric prospective studies are needed to validate our findings. Second, the study is limited by a relatively small sample size to assess clinical outcomes. However, this is a common feature across studies of rare metabolic diseases. It is also important to observe that this is the first study on the role of UA and XO in FD. Our hypothesis of UA elevation in FD is therefore largely based on the current published data on UA and oxidative metabolism in a general population or in patients with a high cardiovascular risk profile. Whether XO pathway plays an important role in LSDs and whether its inhibition may provide any clinical benefit to these patients should be evaluated in future studies.

## Conclusions

In Fabry patients, serum UA levels represent a risk factor for adverse clinical outcomes including cardiovascular and renal morbidity and are associated with all-cause mortality. Increased baseline UA levels in Fabry patients are potentially reflecting the impairment of oxidative and energetic metabolism, endothelial dysfunction and chronic inflammatory response. Because this is the first study indirectly demonstrating an activation of XO in FD, it raises several questions. From a clinical perspective, elevated UA levels seem to represent a cheap and broadly available marker of FD cardiovascular, renal and metabolic involvement. However, this observation should be confirmed by the analysis of larger cohorts of patients from current registries and the use of UA levels in a more complex predictive model ought to be addressed in future studies. As several studies suggested the potential beneficial cardiovascular effects of XO inhibition, its potential role should be evaluated in FD as well.
