# Correction: Uric Acid as a Marker of Mortality and Morbidity in Fabry Disease

**DOI:** 10.1371/journal.pone.0170881

**Published:** 2017-01-20

**Authors:** Daniel Rob, Josef Marek, Gabriela Dostálová, Lubor Goláň, Aleš Linhart

There is an error in the fifth sentence under the subheading “Determination of clinical and laboratory data” in the Materials and Methods. The correct sentence should read: Left ventricular mass index (LVMi) was calculated using the Devereux-modified cube formula based on linear measurements of interventricular septum thickness, left ventricular cavity diameter and posterior wall thickness as assessed by the American Society of Echocardiography (ASE) method and indexed to body surface area (in m^2^) [7].
